# Bacteriophage as a potential therapy to control antibiotic-resistant *Pseudomonas aeruginosa* infection through topical application onto a full-thickness wound in a rat model

**DOI:** 10.1186/s43141-022-00409-1

**Published:** 2022-09-12

**Authors:** Nouran Rezk, Abdallah S. Abdelsattar, Doaa Elzoghby, Mona M. Agwa, Mohamed Abdelmoteleb, Rania G. Aly, Mohamed S. Fayez, Kareem Essam, Bishoy M. Zaki, Ayman El-Shibiny

**Affiliations:** 1 Center for Microbiology and Phage Therapy, Zewail City of Science and Technology, Giza, 12578 Egypt; 2 Center for X-ray and Determination of Structure of Matter, Zewail City of Science and Technology, Giza, 12578 Egypt; 3 Department of Chemistry of Natural and Microbial Products, National Research Centre, Dokki, Giza, 12622 Egypt; 4 Department of Botany, Faculty of Science, Mansoura University, Mansoura, 35516 Egypt; 5 Department of Surgical Pathology, Faculty of Medicine, Alexandria University, Alexandria, 21131 Egypt; 6 Microbiology and Immunology Department, Faculty of Pharmacy, October University for Modern Sciences and Arts (MSA), Giza, 11787 Egypt; 7 Faculty of Environmental Agricultural Sciences, Arish University, Arish, 45511 Egypt

**Keywords:** *Pseudomonas aeruginosa*, Multi-drug resistant (MDR), Wound infection, In vivo, Bacteriophage, Biofilm, Phage characterization, Phage isolation, Immunohistochemical (IHC)

## Abstract

**Background:**

Antibiotic-resistant *Pseudomonas aeruginosa* (*P. aeruginosa*) is one of the most critical pathogens in wound infections, causing high mortality and morbidity in severe cases. However, bacteriophage therapy is a potential alternative to antibiotics against *P. aeruginosa*. Therefore, this study aimed to isolate a novel phage targeting *P. aeruginosa* and examine its efficacy in vitro and in vivo.

**Results:**

The morphometric and genomic analyses revealed that ZCPA1 belongs to the *Siphoviridae* family and could infect 58% of the tested antibiotic-resistant *P. aeruginosa* clinical isolates. The phage ZCPA1 exhibited thermal stability at 37 °C, and then, it decreased gradually at 50 °C and 60 °C. At the same time, it dropped significantly at 70 °C, and the phage was undetectable at 80 °C. Moreover, the phage ZCPA1 exhibited no significant titer reduction at a wide range of pH values (4–10) with maximum activity at pH 7. In addition, it was stable for 45 min under UV light with one log reduction after 1 h. Also, it displayed significant lytic activity and biofilm elimination against *P. aeruginosa* by inhibiting bacterial growth in vitro in a dose-dependent pattern with a complete reduction of the bacterial growth at a multiplicity of infection (MOI) of 100. In addition, *P. aeruginosa*-infected wounds treated with phages displayed 100% wound closure with a high quality of regenerated skin compared to the untreated and gentamicin-treated groups due to the complete elimination of bacterial infection.

**Conclusion:**

The phage ZCPA1 exhibited high lytic activity against MDR *P. aeruginosa* planktonic and biofilms. In addition, phage ZCPA1 showed complete wound healing in the rat model. Hence, this research demonstrates the potential of phage therapy as a promising alternative in treating MDR *P. aeruginosa*.

**Supplementary Information:**

The online version contains supplementary material available at 10.1186/s43141-022-00409-1.

## Background

*Pseudomonas aeruginosa* (*P. aeruginosa*) is a Gram-negative, rod-shaped, aerobic, opportunistic pathogen responsible for 10% of hospital-acquired infections worldwide [1]. *P. aeruginosa* is a primary cause of pneumonia, wound infections, urinary tract infections, and bacteremia [2]. Antibiotics used to treat *P. aeruginosa* infections became ineffective due to worldwide antimicrobial resistance. It was estimated in 2019 that about 5 million deaths were because of antibiotic-resistant bacterial infections [3]. Moreover, the World Health Organization (WHO) indicated that antimicrobial-resistant bacterial infections could kill approximately 10 million people per year by 2050 [4]. In addition, WHO has classified it as a critical pathogen that requires immediate attention to develop new antibiotics [1].

*P. aeruginosa* has a high ability to develop resistance against a wide range of antibiotics, including fluoroquinolones, aminoglycosides, β-lactams, and different disinfectants. This is because it contains intrinsic and acquired mechanisms. The intrinsic resistance of *P. aeruginosa* includes reduction of membrane permeability, overexpression of efflux pumps that pump antibiotics out of the cell, and the production of antibiotic-degrading enzymes, including AmpC β-lactamases and aminoglycoside-modifying enzymes [5, 6]. Moreover, biofilm development is considered one of the primary resistance mechanisms in *P. aeruginosa*. *P. aeruginosa* biofilms are critical in protecting bacteria from host immune responses and are up to 1000 times more resistant to antibiotics than planktonic cells [7, 8]. Multidrug-resistant (MDR) *P. aeruginosa* has been linked to nosocomial infections and outbreaks in burn and intensive care units [9–11] and is a growing cause of hospital-acquired infections [12]. *P. aeruginosa* is also considered a severe threat by the Centers for Disease Control and Prevention (CDC) because of its high mortality rate [13]. Moreover, Egypt has the highest MDR *P. aeruginosa* prevalence (75.6%) in the Middle East (MENA) region [14].

Skin is the first defense barrier against bacterial infections, and any damage to the skin allows pathogens to enter the body, resulting in sepsis and death [15]. Wounds and skin damage can be induced by chemical and physical agents and diseases like diabetes [16]. Wound infection is one of the most common nosocomial infections with a high mortality rate [17]. *P. aeruginosa* is the most frequent driver of wound infections, which colonizes the wound site followed by expansion into internal organs through the bloodstream that ends with skin necrosis and serious complications and, in severe cases, can be life-threatening [16, 18, 19]. Moreover, *P. aeruginosa* is a significant cause of delayed wound healing. A meta-analysis study showed that 78.2% of biofilms in chronic non-healing wounds were associated with poor wound healing [20, 21]. According to Ijaz et al., MDR *P. aeruginosa* was found in 58.6% of clinical samples from wounded patients [22]. Therefore, better therapeutic and prophylactic treatments for combating MDR *P. aeruginosa* wound infections are required.

Phage therapy is one of the promising alternatives to antibiotics, which utilizes naturally occurring phages that are considered part of the human microbiome [23, 24] and have high specificity towards their bacterial hosts, including biofilm-forming and antibiotic-resistant bacteria [25–27]. Many research studies have highlighted the use of phages to control biofilm-forming bacteria, including *P. aeruginosa* and demonstrated the ability of phages to eradicate the *P. aeruginosa* infections [28, 29] and biofilm-forming bacterial cells [16, 30–33]. However, previous studies have focused mainly on the treatment of cystic fibrosis caused by *P. aeruginosa* by phages in vivo [29, 34–36]*.* Moreover, using phage therapy against MDR *P. aeruginosa* in wound infection shows high efficacy and safety through topical and oral administrations [29, 37–39]. Hence, this study aimed to isolate and characterize a novel phage against MDR *P. aeruginosa* from Egyptian clinical isolates and study its efficacy in vitro and on infected wounds using a rat model through single and multiple doses.

## Methods

### Bacterial characterization

#### Bacterial growth conditions

Fifty clinical wound swabs were collected from patients in Egyptian hospitals infected with P. aeruginosa and given as a gift to the Center for Microbiology and Phage therapy at Zewail City for Science and Technology. First, the clinical isolates were streaked on Cetrimide agar (Oxoid, England), and P. aeruginosa was identified according to the standard morphology on cetrimide agar that appears fluorescent green. Then, five colonies were picked for purification and stored in Tryptic Soy Broth (TSB; Oxoid, England) containing 20% (v/v) glycerol for storage at −80 °C. Before each experiment, fresh bacterial cultures were prepared by inoculating a single colony from Cetrimide agar into 1 mL of TSB in 1.5-mL Eppendorf and incubating for 16 h at 37 °C with shaking at 200 rpm. All methods were performed following the relevant guidelines and regulations.

#### Detection of *P. aeruginosa* and virulence genes using polymerase chain reaction (PCR)

According to the manufacturer’s instructions, the bacterial DNA was extracted by QIAamp DNA Mini Kit (Qiagen, Germany). Seven virulence genes were used to confirm the pathogenicity and antibiotic resistance of the bacterial strains. Briefly, the total volume of each reaction was 25 μL containing 12.5 μL of 2x master mix (Thermo Scientific), 1 μL from each stock primer, 2 μL of bacterial DNA, 0.5 μL of 50 mM MgCl_2_, and 8 μL of distilled water. Afterwards, the products were run through 1% (w/v) agarose gel (Sigma-Aldrich, USA) to determine the size of the PCR product. All the primer sequences, the predicted sizes, and annealing temperatures are shown in Table 1.

**Table 1:** Primer set sequences for the seven virulence and resistance genes

Primer	Sequence	Product size (bp)	Annealing temperature (°C)	References
***ETA***	F: GACAACGCCCTCAGCATCACCAGC	396	60	[40]
	R: CGCTGGCCCATTCGCTCCAGCGCT			
***PhzI***	F: CATCAGCTTAGCAATCCC	392	50	[41]
	R: CGGAGAAACTTTTCCCTC			
***PhzII***	F: GCCAAGGTTTGTTGTCGG	1,036	51	[41]
	R: CGCATTGACGATATGGAAC			
***bla-TEM***	F: ATGAGTATTCAACATTTCCG	867	50	[42]
	R: CTGACAGTTACCAATGCTTA			
***OprL***	F: CGTGCGATCACCACCTTCTA	171	52.5	Designed for this study
	R: CTCGCCCAGAGCCATATTGT			
***ExoS***	F: GCCTTGTCGAGTCCCTTCAA	345	53.5	Designed for this study
	R: GCTTCAGCAGAGTCCGTCTT			
***PlcH***	F: CCTGACTTCGCTGTTCGACT	680	53.5	Designed for this study
	R: CCTGGTTCGGTTCGAGTTCA			

#### Antimicrobial susceptibility testing

The antibiotic profile of fifty *P. aeruginosa* isolates was investigated using the disc diffusion method [35]. The tested antibiotics were ciprofloxacin (CIP; 5 μg), gentamicin (CN; 10 μg), imipenem (IPM; 10 μg), cefepime (Feb; 30 μg), levofloxacin (LEV; 5 μg), amikacin (AK;30 μg), piperacillin (PRL; 10 μg), and meropenem (MEL; 5 μg) (Oxoid, UK). The diameter of the clear zone was measured, and the results were interpreted based on the Clinical and Laboratory Standards Institute (CLSI) [43].

#### 16S rRNA gene sequencing

A second PCR reaction was performed to identify the bacterial strain *P. aeruginosa* (*P.s* 12), the primary host for further phage isolation and other studies using 16S rRNA gene sequencing. The PCR reaction volume was 50 μL, including 25 μL of 1x Master Mix (Thermo Scientific, USA), 5 μL of DNA sample was extracted by QIAamp DNA 93 Mini Kit (Qiagen, Germany), and 18 μL of nuclease-free water, and 1 μL of the forward primer (5′-AGAGTTTGATCCTGGCTCAG-3′), 1 μL of the reverse primer (5′-TACGGYTACCTTGTTACGACTT-3′). PCR program was 94 °C for 3 min, 30 cycles at 94 °C for 30 s, 55 °C for 30 s, 72 °C for 1 min, and 72 °C for 10 min. In addition, 1.5 % (v/w) of the agarose gel was used in gel electrophoresis for the separation of the PCR product, and gel purification was performed of 16s rRNA gene using QIAEX II Gel Extraction Kit (QIAGEN, Germany) as described in the manufacturer’s protocol [41].

The 16S rRNA sequence was determined with a model 373A automated fluorescent-DNA sequencer (Applied Biosystem, USA). The nucleotide sequence of the 16s rRNA gene that was obtained was processed through Finch TV software (https://digitalworldbiology.com/FinchTV). BLASTN (Basic Local Alignment Search Tool, (http://www.ncbi.nlm.nih.gov/BLAST/Blast.cgi) against the 16S ribosomal RNA database was performed to identify the isolated strain [44]. The 16S rRNA sequence was deposited in the NCBI GenBank under accession number OL375153.

### Phage isolation, purification, and propagation

More than ten sewage samples were taken from raw sewage, primary and secondary treatment stages from two different sewage treatment stations in Giza, Egypt. The samples were used for phage isolation using enrichment techniques [45, 46]. Briefly, 1mL of overnight culture from *P.s* 12 was added to 9 mL of sewage samples and incubated at 37 °C for 4h, 1% of chloroform was added to the mixture and centrifuged at 5000 rpm for 20 min, and the supernatant was retained. Afterwards, a spot assay was used by mixing 100 μl of exponential-phase bacterial host culture with 4 mL of soft agar (0.3% w/v agar), which was held at 55 °C, used at a suitable temperature between 45 and 50 °C, then poured into a TSA plate to create a bacterial layer. Ten microliters of each supernatant test sample was spotted on the bacterial layer, and the plates were incubated at 37 °C for 24 h. After incubation, if any clear zones or plaques were observed on the plates, then the plaques were picked using a sterile pipette tip, suspended in 100 μl of Gelatin-SM buffer [5.8 g NaCl, 2.0 g MgSO_4_·7H_2_O, 50 mL 1 M Tris-HCl pH 7.4, in 1-liter dH_2_O], and stored at 4 °C for 4 h. Subsequently, a tenfold serial dilution of each plaque and spot assay was made on the bacterial host and repeated several times to get a single purified phage. Finally, 10 mL of the bacterial host culture was infected with 100 μl of a single phage to increase the number of Plaque Forming Unit (PFU)/mL, which were measured by spot assay of decimal dilutions.

### Phage characterization

#### Pulsed-field gel electrophoresis (PFGE)

PFGE analysis was used to estimate the genome size of the isolated phage as described by Lingohr et al. [47] with slight modifications. Briefly, 100 μl of phage suspension (10^9^ PFU/mL) was mixed with 100 μl of 1.4% of plug agarose. After solidification, the plugs were immersed in lysis buffer (1 mg/mL Proteinase K [ThermoFischer Scientific, USA]); 0.2% w/v SDS [Sigma Aldrich, Gillingham, UK]; and 100 mM EDTA; 1% w/v N-Lauryl sarcosine [Sigma Aldrich, Gillingham, UK] and incubated at 55 °C for 18 h. After incubation, the plugs were washed and added to the PFGE 1.5% agarose gel and Lambda PFG Ladder (Biolabs, UK) was used. The PFGE was run for 18 h at 200 V (6 V/cm) with a 30- to 60-s switch time using a Bio-Rad CHEF DRII system (Biorad, USA). For imaging, 5 μl of ethidium bromide (Carl Roth, Germany) was added to the distilled water, washed, and analyzed under UV light at the Gel Dock imaging system (Biorad, USA).

#### Host range analysis

The lytic activity of the isolated phage against fifty clinical isolates of *P. aeruginosa* was investigated using the spot assay in triplicate as previously described [46]. The appearance of clear zones (bacterial lysis) confirmed the susceptibility of bacterial strains to the phage in the spotting area.

#### Phage DNA sequencing

Genomic DNA was extracted from the isolated phage with 10^10^ PFU/mL lysates. According to the manufacturer’s instructions, the DNA extraction was performed using proteinase K treatment (100 μg/mL in 10 mM EDTA pH 8), followed by resin purification using the Wizard DNA extraction kit (Promega, UK). The nucleotide sequencing was performed using the Illumina MiSeq platform. Nextera XT DNA Library Preparation Kit was used to prepare the DNA library (Illumina, Cambridge, UK). The paired-end DNA reads were evaluated for accuracy using FASTQC [48]. Moreover, low-quality bases were trimmed using PRINSEQ. Cleaned reads were de novo assembled using SPAdes [49] with multiple K-mers: 21, 33, 55, 77, and 99 . Genome assembly was checked for quality using QUAST [50]. BLASTn against the NCBI Nucleotide database was used to identify closely related phages. Then, the top-matched phages were imported into MEGA-X [51] to draw a phylogenetic tree using CLUSTAL-W aligner [52] and the best Maximum likelihood fit model (GTR: general time reversible substitution model, G: Gamma distributed among sites). Open-reading frames were predicted using ORF finder. The predicted ORFs were compared against the NCBI non-redundant protein sequences (nr) database using BLASTp to identify putative coding sequences (CDSs). The predicted putative genes were compared to those predicted through PHASTER [53]. The annotated genome of the isolated phage (ZCPA1) has been deposited in the NCBI GenBank under the accession numbers OL597541:OL597590. The genetic map focusing on putative coding genes was generated using SNAPGene (GSL Biotech; available at https://www.snapgene.com/; Access date: 27 July 2021).

#### Physical stability of phage

The thermal stability of the ZCPA1 with a high titer (10^9^ PFU/mL) was performed by incubating tubes containing the phage suspended in SM buffer at 37, 50, 60, 70, and 80 °C for 1 h. After incubation, the phage was enumerated through ten-fold serial dilution and spotted in triplicate by the spot test assay as previously described. Furthermore, the pH stability was investigated using various test tubes containing deionized water with a pH range (2–11) adjusted using HCl and NaOH, and incubated for 1 h [27]. Then, the phage titer was calculated by the spot test assay. In addition, the phage susceptibility against UV inactivation was determined by exposing the phage suspended in SM buffer directly to UV radiation (λ=253 nm) for 1 h. A sample was taken for phage enumeration at 10-min intervals using spot test assay in triplicate.

#### Biofilm clearance assay

The antibiofilm activity of phage was investigated against established (*P.s* 12) biofilm. The methodology was described in previous studies [54, 55]. Briefly, the bacterial culture grown in TSB was supplemented with 0.1% glucose for biofilm enhancement, placed in a 96-well plate as six replicates and left for 48 h to form biofilm at 37 °C. After incubation, phage was added to the formed biofilm with different multiplicity of infections (MOIs): 0, 0.01, 0.1, 1, 10, and 100, and left for another 24 h. Then, the plate was washed to remove any unattached cells. A 1% crystal violet was added to the wells for staining, and 30% glacial acetic acid was used for the quantitative assay using FLUOstar® Omega Microplate Reader at OD_540_ (BMG LABTECH, Germany).

#### One-step growth curve

As previously described by Taha et al. [56], a one-step growth curve was conducted to determine the different stages of the phage replication cycle. The bacterial culture (*Ps.* 12) was grown in TSB at 10^5^ colony forming unit (CFU/mL), infected by the isolated phage at MOI 1, and incubated at 37 °C for 4 h. During the incubation time, 200-μL aliquots were withdrawn at various time points (0, 10, 20, 30, 45, 60, 90, 120, 180, and 240 min) and divided into two 100-μL samples. A 1% chloroform was added to one of them to determine the eclipse period through releasing the intracellular phages, and another one was left without chloroform. Both samples were tenfold serially diluted, and the phage titer was enumerated by the spot test assay as previously described.

#### Time-killing curve

The bacterial reduction was determined using phage ZCPA1 at different MOIs (0.1, 1, 10, 100). One hundred eighty microliters of bacterial culture at 10^6^ CFU/mL combined with 20 μL of the phage suspension with different MOIs (0.1, 1, 10, 100). Six replicates were done using 96-well plate. The FLUOstar® Omega plate reader was used for monitoring the OD_600_ changes, and the data were collected at 5 min intervals for 4 h at 37 °C.

#### Morphological characterization by transmission electron microscopy (TEM)

The phage was imaged using TEM JEOL 1230 in the faculty of science, Alexandria University, Egypt. The phage (10^9^ PFU/mL) was prepared in SM buffer and submerged on Formvar carbon-coated copper grids (Pelco International), then stained with 2% phosphor tungstic acid (pH 7.0) and subjected to drying before TEM examination.

### Assessment of phage against *P. aeruginosa*-infected wound rat model

#### Surgical procedures of full-thickness wound model

The solution of phage suspension can be applied locally to the wounded skin area to examine its healing potential on the infected full-thickness excision skin wound in a rat model. The animal experiments were certified by the Animal Care Committee of the Alexandria University (ALEXU-IACUC), with an authorized acceptance for the surgical procedures (AU-IACUC-14/2100601-3-6). This study is reported following ARRIVE guidelines (Animal Research: Reporting of In Vivo Experiments) (https://arriveguidelines.org).

Twenty-five Wistar male rats (8 weeks old), weighing approximately 180–200 g, were selected in the in vivo animal study. The rats were housed individually in a separate stainless-steel cage allowing free access to a standard laboratory diet and mineral water ad libitum. According to formerly published studies, the surgical procedures for the full thickness excision wound were carried out [57–59]. First, the animals were anaesthetized by an intramuscular injection of xylazine (10mg/kg) and ketamine (60 mg/kg). Then, the back hair in the dorsal areas was removed using an electric shaver. A rounded wound was formed, with a diameter of 15 mm on the dorsal area of the rats using dissecting scissors and sterile forceps. The animals were randomly categorized into five groups with five rats. The first group includes applied sterile gauze (no wound infection) as a positive control. Wound infection was completed by inoculating the wound with *P.s* 12 (1 × 10^7^ CFU/mL) for 2 h before applying the treatment. Both 2nd and 3rd groups were applied sterile gauze as a negative control and gentamicin cream as an antibiotic treatment, respectively. The 4th and 5th groups were applied multi-dose and single-dose phage with 1 × 10^9^ PFU/mL, respectively. A swab for all groups was taken to calculate the bacterial count inhabiting the wound on the 3rd, 7th, 10th, and 14th days post-surgery. The gentamicin and phage (for the multi-dose group) were reapplied each time. Moreover, the wound was rated and photographed via a digital camera. The wound area was estimated using a digital caliper, and the percentage wound closure rate was calculated from the following equation [60, 61]:

$$\mathrm{Wound}\;\mathrm{closure}\;(\%)\:=\:\left[1\:-\:\left(\mathrm{Wound}\;\mathrm{area}\;\mathrm{on}\;\mathrm{given}\;\mathrm{day}/\mathrm{Wound}\;\mathrm{area}\;\mathrm{on}\;\mathrm{day}\;0\right)\right]\:\times\:100.$$

#### Histological examination

On the 17th day, the rats were sacrificed, followed by the removal of the tissue from the wound bed and its surrounding healthy skin to assess the skin wound healing. For histopathological investigations, the tissue was fixed in 10% formaldehyde and embedded in paraffin blocks. The skin sections were stained with hematoxylin and eosin (H&E) and examined under a light microscope (Leica, Germany). A histological examination of the whole wound area was done. The mean value of the percentage of the fibrous tissue, the granulation tissue, and the epidermal thickness was quantified using ImageJ, v1.53 (Maryland, USA). The skin appendages, hair follicles and sebaceous glands, were scored (no skin appendages in wound area= score 1, few <5/wound area = score 2, and ≥5/wound area = score 3). The skin sections were also stained with Masson trichrome staining. Image color deconvolution v1.53 (Maryland, USA) was used to quantify the percentage area of fibrosis in each specimen [62, 63].

#### Immunohistochemical (IHC) staining and interpretation

IHC of all sections was done using the Avidin-Biotin-Peroxidase method [64]. CD45 (ready to use primary antibody, mouse anti-human, monoclonal antibody, P0042; Leica Biosystems, USA) and α-SMA (ready to use primary antibody, mouse anti-human, monoclonal antibody, P0943; Leica Biosystems, USA) were used to stain the lymphocytes, blood vessels, and myofibroblasts respectively. The antibodies were added to each section using the Bond-Max fully automated immunostainer (Leica Biosystems, USA). Positive control for each primary antibody using tonsil and leiomyoma was added in each run. Negative control omitting the primary antibody was also added in each run. The IHC quantification of CD45 and α-SMA was performed on each slide using the quantitative-image analysis (Leica microsystems, Switzerland).

### Statistical analysis

All experiments were conducted in triplicates, and the results were illustrated in the form of mean ± standard deviation (SD). In this study, GraphPad Prism v5 software was used to generate graphs and perform all statistical analyses. Both Student’s *t*-test (two-tailed) and ANOVA tests were used during the work to evaluate the significance *p* < 0.05.

## Results

### Bacterial characterization

Antibiotic susceptibility is classified into sensitive, intermediate, and resistant (Additional file 1: Table S1). The majority of the bacterial strains showed a high resistance pattern to fluoroquinolones (FQs), with 65 % of levofloxacin resistance and 59 % of ciprofloxacin resistance. These findings indicated that *P. aeruginosa* was more resistant to levofloxacin than ciprofloxacin. Moreover, the *P. aeruginosa* isolates exhibited high resistance to beta-lactams, including 67% resistance to meropenem. Gentamicin demonstrated a high resistance pattern at 63%. Accordingly, 29 of the tested *P. aeruginosa* isolates were MDR bacteria.

Moreover, seven virulence and antibiotic-resistance genes (*Exo A*, *Exo S*, *Phz I and II*, *bla-TEM*, *OprL* and *plcH*) have been identified in the ten resistant *P. aeruginosa* isolates tested using PCR. Furthermore, using BLASTn, the 16S rRNA sequence of *P.s* 12 was found to be 99% identical to *P. aeruginosa* strain B19 16S ribosomal RNA gene, the partial sequence with GenBank Acc. No. MZ425417.1.

### Phage characterization

#### The ZCPA1 phage morphology

**Fig. 1:**
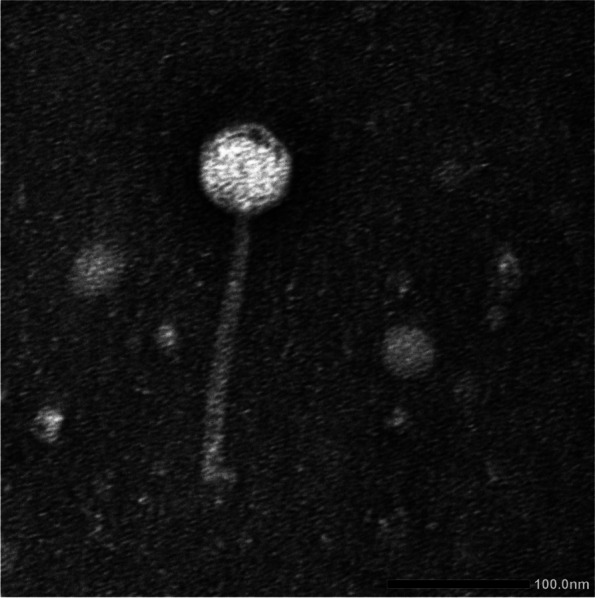
Transmission electron microscopic image of phage ZCPA1

According to the morphology of the phage, it belongs to the *Siphoviridae* family. The phage consists of an icosahedral head and a long noncontractile flexible tail. The phage head-to-tail length ratio was similarly typical of the *Siphoviridae* family, with a 66.80-nm width of icosahedral head and 188.54 nm long tail (Fig. 1).

#### Host range analysis

The lytic activity of the phage ZCPA1 against different resistant bacterial strains was evaluated. The ZCPA1 exhibited a broad host range with lytic activity against 58% of the tested *P. aeruginosa* clinical isolates (Table 2).

**Table 2:** The lytic activity of phage ZCPA1 against 50 clinical isolates of *P. aeruginosa* (*P.s* 1 to *P.s* 50)

Bacterial strain	ZCPA1	Bacterial strain	ZCPA1	Bacterial strain	ZCPA1	Bacterial Strain	ZCPA1	Bacterial strain	ZCPA1
*P. s* 1	+	*P. s* 11	−	*P. s* 21	+	*P. s* 31	+	*P.s* 41	−
*P. s* 2	−	*P. s* 12	+	*P. s* 22	+	*P. s* 32	+	*P.s* 42	−
*P. s* 3	+	*P. s* 13	+	*P. s* 23	+	*P. s* 33	+	*P.s* 43	−
*P. s* 4	−	*P. s* 14	−	*P. s* 24	+	*P. s* 34	+	*P.s* 44	−
*P. s* 5	−	*P. s* 15	+	*P. s* 25	+	*P. s* 35	−	*P.s* 45	−
*P. s* 6	−	*P. s* 16	+	*P. s* 26	+	*P. s* 36	+	*P.s* 46	−
*P. s* 7	−	*P. s* 17	+	*P. s* 27	+	*P. s* 37	+	*P.s* 47	−
*P. s* 8	−	*P. s* 18	+	*P. s* 28	+	*P. s* 38	+	*P.s* 48	−
*P. s* 9	−	*P. s* 19	+	*P. s* 29	+	*P.s* 39	+	*P.s* 49	−
*P. s* 10	−	*P. s* 20	+	*P. s* 30	+	*P.s* 40	−	*P.s* 50	−

+ indicates lysis, and − indicates no lysis

#### Physical stability of the phage

**Fig. 2:**
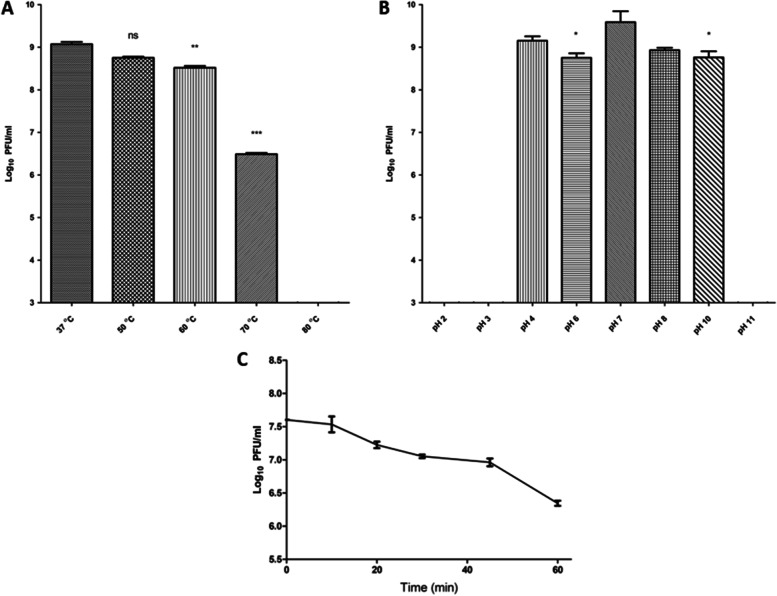
The physical stability of the phage ZCPA1. **A** Phage viability at different temperatures, **B** phage viability at different pH values, and **C** phage viability under UV light (*λ* = 253 nm). ns stands for no significance, *P < 0.05, **P < 0.01, and ***P < 0.001

To use ZCPA1 in therapy, phage stability was examined against various environmental conditions, including heat, pH, and UV. The ZCPA1 exhibited thermal stability at 37 °C with 10^9^ PFU/ mL, then decreased gradually to 10^8^ PFU/mL at 50 °C and 60 °C. At the same time, it dropped significantly to 10^6^ PFU/mL at 70 °C, and the phage was undetectable at 80 °C (Fig. 2A). Moreover, phage ZCPA1 exhibited no significant titer reduction at a wide range of pH values (4–10) with maximum activity at pH 7, and the phage performance was changed after exposure to extreme pH conditions as its infectivity was significantly inactive at pH 2, 3, and 11 (Fig. 2B). In addition, the phage showed resistance to UV inactivation for 45 min under UV light with one log reduction after 1 hour (Fig. 2C).

#### The phage genome

**Fig. 3:**
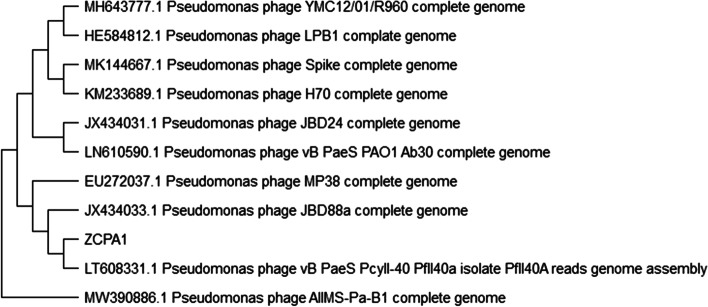
Phylogenetic relationships between phage ZCPA1 and BLASTn top-matched phages. The closely related phage is *Pseudomonas* phage vB_PaeS_PcyII-40_PfII40a isolate genome (GenBank Acc. No. LT608331.1)

The genome of phage ZCPA1 has been sequenced and deposited in the GenBank database (GenBank Acc. No. OL597541:OL597590). Phage ZCPA1 contains a double-stranded DNA genome of 46 kbp, which also agreed with the PFGE results. The phage ZCPA1 has an overall G + C content of 64.04%. Moreover, BLASTn analysis confirmed that ZCPA1 is a member of the *Siphoviridae* family, in the order of *Caudovirales*. Assembly parameters were as follows: contigs# 476, N50 1648, L50 8, N’s per 100 kb 0. BLASTn alignment and phylogenetic tree showed that *Pseudomonas* phage vB_PaeS_PcyII-40_PfII40a isolate genome, chromosome (GenBank Acc. No. LT608331.1), was closely related to the genome of ZCPA1 (Fig. 3). The annotated genes were manually curated and listed in Additional file 1: Table S2. The open reading frames (ORFs) prediction applying the standard genetic code identified ninety putative protein-coding genes. The functional genes of phage ZCPA1 are shown in the genetic map (Additional file 1: Figure S1). Among these, forty predicted proteins have assigned functions, involving cell lysis proteins, DNA replication/transcription/repair structural proteins, and DNA packaging proteins. ZCPA1 has fifty ORFs on the leading strand and forty ORFs on the complementary strand.

#### In vitro characterization of phage ZCPA1

**Fig. 4.**
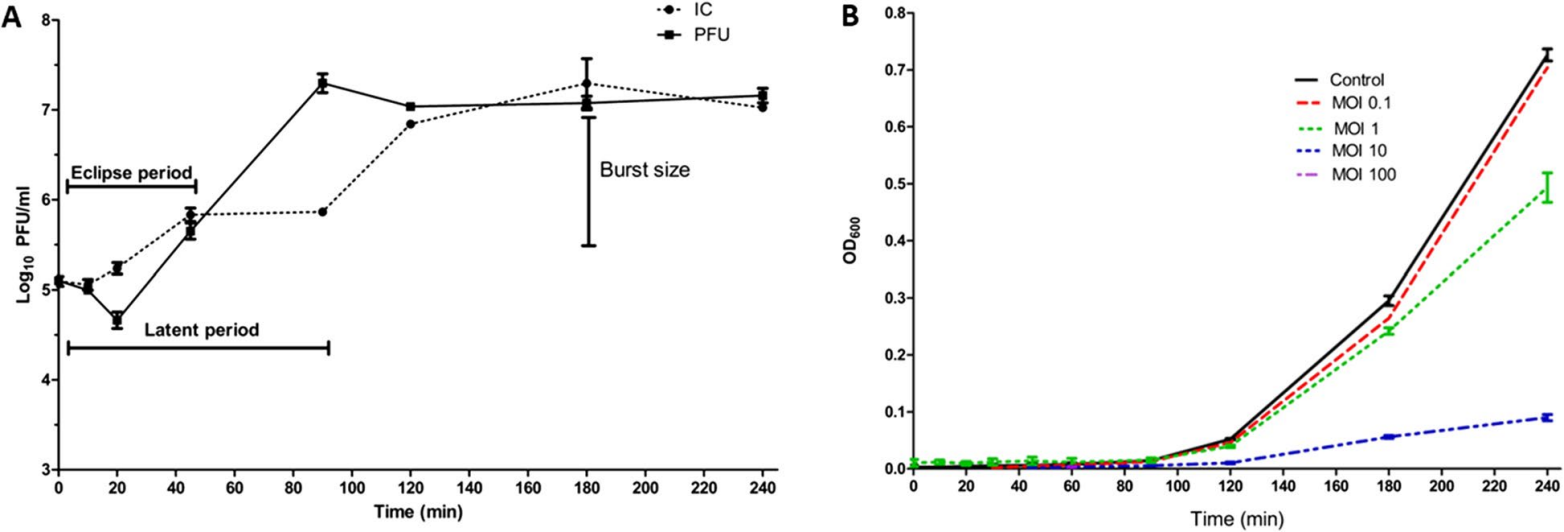
**A** One-step growth curve of ZCPA1 at MOI 1. IC indicates phage infective centers, and PFU indicates plaque forming unit. **B** Phage killing curve at different MOIs over 240 min

A one-step growth curve determined the replication cycle of the phage ZCPA1 (Fig. 4A). The IC refers to the phage infective centers, indicating the number of free virions released from the bacterial cells without the addition of chloroform, while PFU indicates the number of virions inside and outside the bacterial cell due to chloroform addition. The latent phage period was estimated to be about 90 min, in which the phage virions were naturally released from the bacterial host. The eclipse period of the phage was about 45 min, which indicates the required time for viral particles to be synthesized and assembled since the bacterial lysis was aided by chloroform addition. The burst size was calculated at about 68 virions per single bacterium.

The lytic activity of phage against *P.s* 12 was tested at different MOIs (0.1, 1, 10, 100). During the first 3h, the bacterial turbidities at OD_600_ were 0.295 ±0.02 for bacterial control, 0.264 ±0.03 for MOI 0.1, 0.242 ±0.01 for MOI 1, 0.056 ±0.005 for MOI 10, and around 0 for MOI 100. However, after 4 h-incubation, the media turbidity changed to OD_600_ values of 0.72, 0.70, 0.493, 0.09, and around 0 for the control bacteria (bacteria without phage treatment) and the bacteria treated with phage at various MOIs, in the same order as described above. (Fig. 4B). As a result, the optimum MOI of the phage ZCPA1 is 10.

#### Biofilm clearance assay

**Fig. 5:**
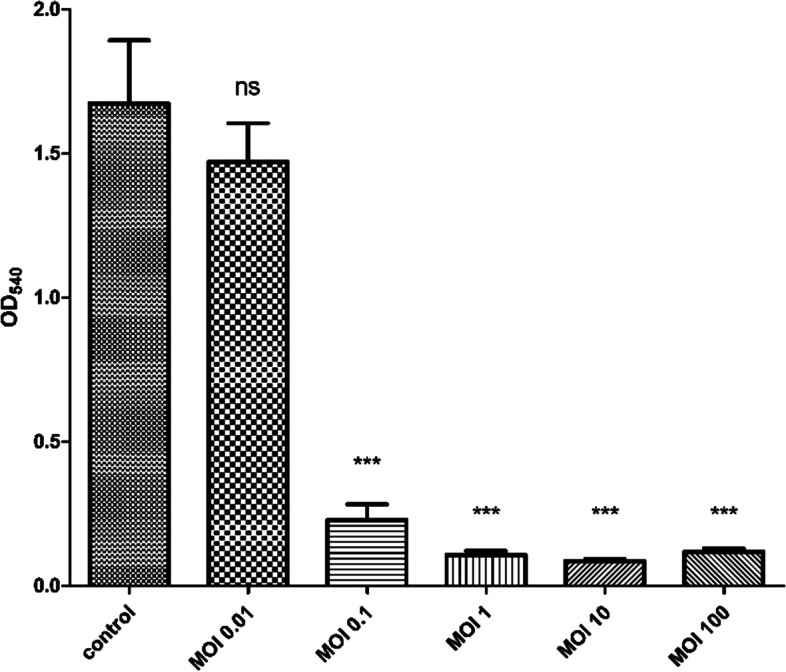
The impact of different MOIs on biofilms clearance. ns stands for no significance, *P < 0.05, **P < 0.01, and ***P < 0.001

Besides, the phage ZCPA1 exhibited a high level of reduction in the *P.s* 12 biofilm biomass, starting from low MOIs of 0.1 to high MOIs of 100. At OD_540_, the established biofilm biomass by *P.s* 12 was 1.67, 1.47, 0.23, 0.11, 0.09, and 0.12 for the -ve control and phage with MOI of 0.01, 0.1, 1, 10, and 100, respectively (Fig. 5).

### In vivo wound healing

#### Photodocumentary analysis and wound healing percentage

**Fig. 6:**
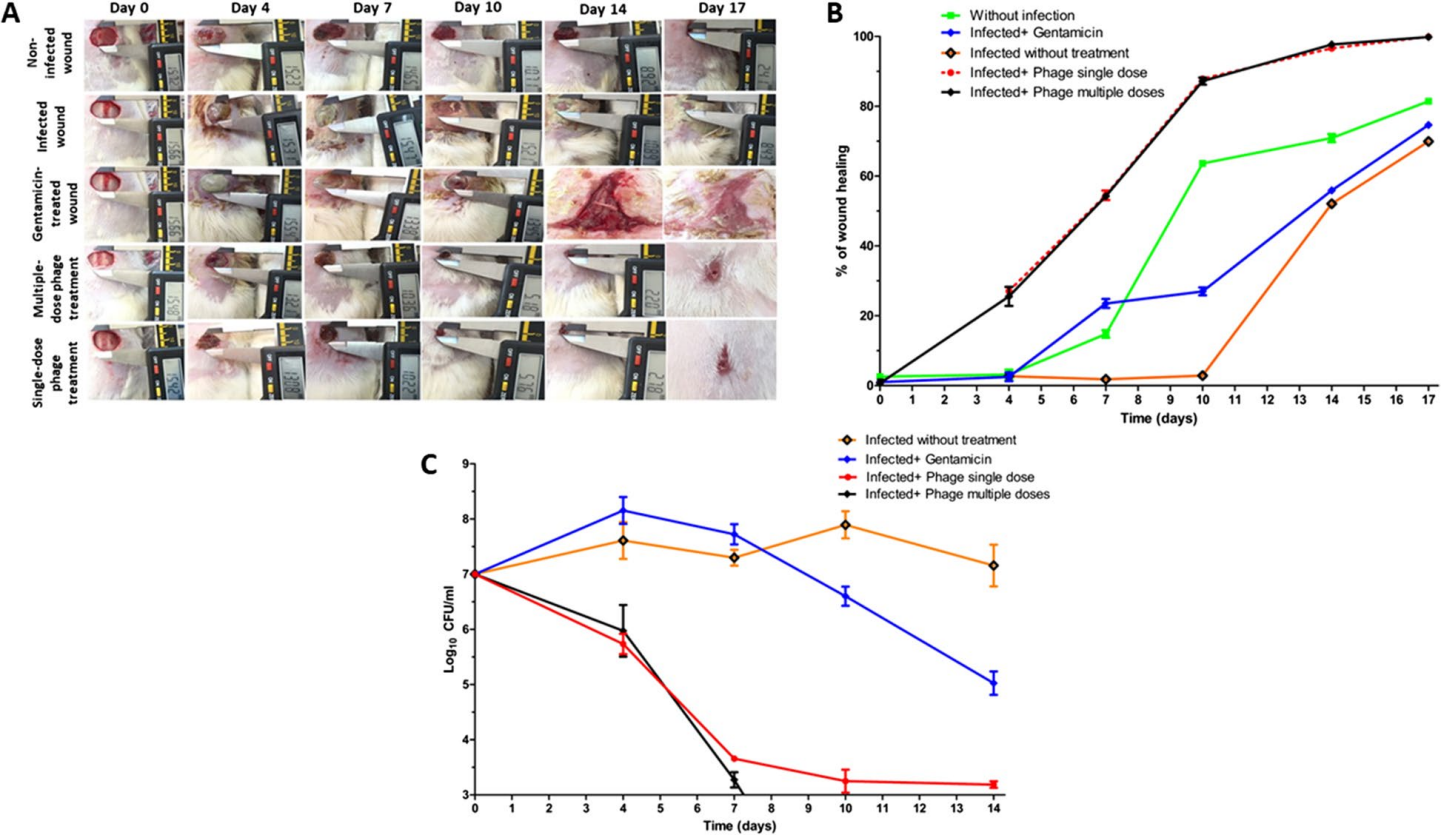
The nontreated and noninfected wound (positive control; group 1), nontreated infected wound (negative control; group 2), infected wound treated with gentamicin antibiotic (group 3), infected wound treated with multiple phage doses (group 4), and the infected wound treated with single phage dose (group 5) from day zero till day 17. **A** Morphometric analysis of full-thickness excision infected wounds in rats by millimeter (mm) at a fixed focal distance. **B** Percentage of wound closure throughout the 17 days. **C** Enumeration of bacteria in the wound for the four groups throughout 14 days

The wound closure percentage, bacterial count, and histopathological analysis throughout 17 days indicate that the phage ZCPA1 is a promising antibacterial agent against the MDR *P. aeruginosa* with high-quality wound healing in a full-thickness wound infection in a rat model. Figure 6A and B display the percentage of wound closure, which is critical for the successful repair of wound tissues. By day 17, both phage-treated groups showed prompt epithelialization of the wound area, evidenced by a higher percentage of wound closure of 99.84% and 99.93% for the groups in which phage was applied in single and multiple doses, respectively. On the other hand, the negative control group that applied sterile gauze showed a lower percentage of wound closure (69.66%). In addition, the gentamicin-treated group exhibited expansion and enlargement of the wound area, resulting in non-healing wounds with purulence and foul odor. The bacterial count of the wounds for all the groups was investigated throughout the 14 days. According to Fig. 6C, the detection limit was 10^2^ CFU/mL. The single phage dose showed a 4 log_10_ reduction, while multiple phage doses exhibited more than 4 log_10_ reductions in the total bacterial count. In contrast, the gentamicin-treated wound showed a 2 log_10_ reduction compared to the positive control.

#### Histopathological analysis of wound healing

**Fig. 7:**
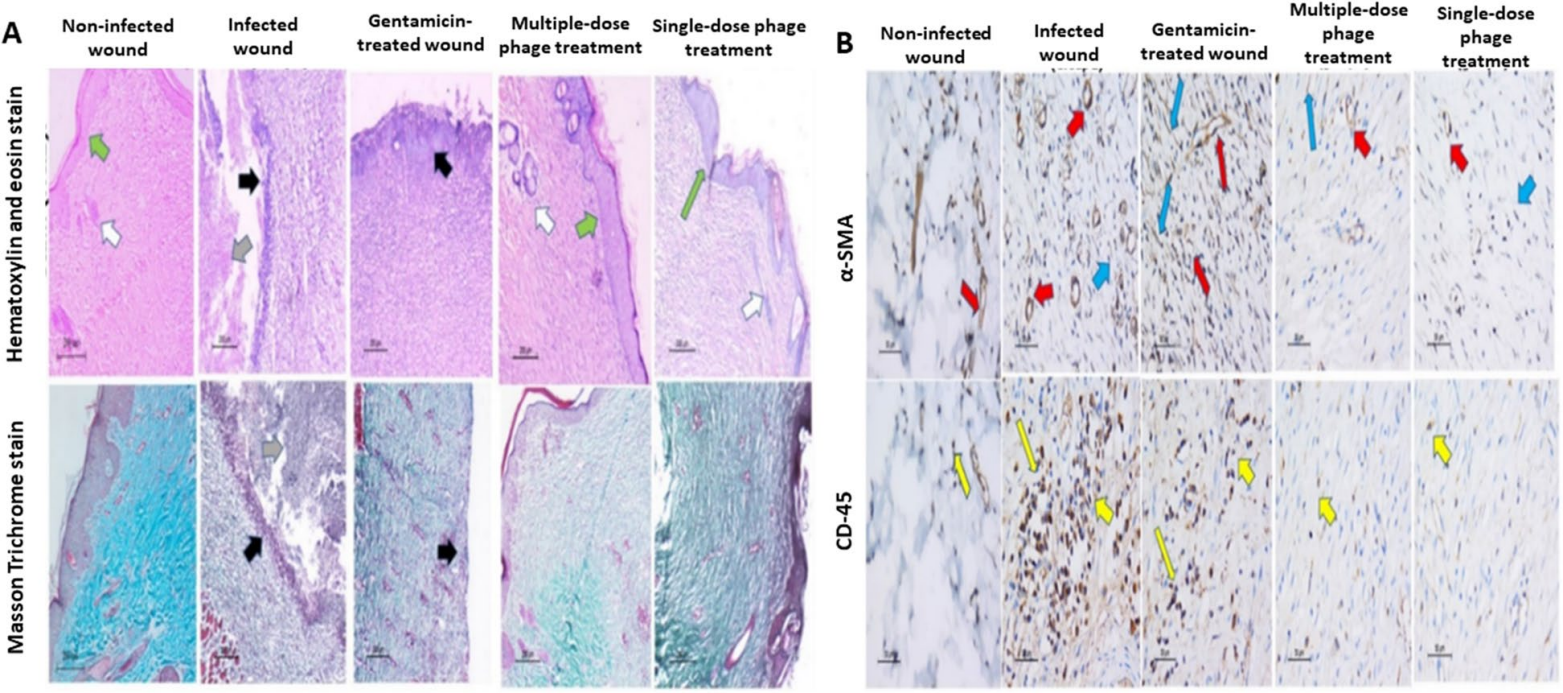
Representative histopathological images of wound tissues on day 17. **A** Hematoxylin and eosin (H&E) staining, ×100, Masson Trichrome stain, ×100, and **B** IHC by using α-SMA and CD-45, ×400 (skin adnexa; white arrow, skin ulceration; black arrow, epidermis; green arrow, blood vessels; red arrow, myofibroblasts; blue arrow, lymphocytes; yellow arrow)

Figure 7 shows skin specimen from the positive control group (group 1) exhibited epidermal re-epithelialization (green arrow) 52.3±0.4 μm with the regeneration of the appendages ≥5/wound area (score 3) (evidenced by H&E staining ×100). The dermis was formed predominantly of collagen fibers and fibrous tissue 95.3±3.2% (remodeling phase) (highlighted by the Masson’s trichrome stain ×100). The granulation tissue was minimal and formed of few newly formed blood vessels 6±2.1/HPF, few myofibroblasts 7±1.2/HPF (highlighted by the immunohistochemistry for α-SMA, ×400), and few lymphocytes 6±2.0/HPF (highlighted by the immunohistochemistry for CD-45, ×400). Skin specimen from the negative control group (group 2) showed extensive epidermal ulceration and crust formation 34±3.2, lacking skin appendages (H&E staining ×100). The dermis showed an excessive number of inflammatory cells (inflammatory phase) and a minimal amount of fibrous tissue (highlighted by the Masson’s trichrome stain ×100) 2.1±0.3%. The granulation tissue was formed of newly formed blood vessels 25±2/HPF, myofibroblasts 20.4±2.5/HPF (highlighted by the immunohistochemistry for α-SMA, ×400), and an increased number of lymphocytes 78±4.2/HPF (highlighted by the immunohistochemistry for CD-45, ×400). Skin specimen from gentamicin treated group (group 3) showed epidermis ulceration, lacking skin appendages (evidenced by H&E staining ×100). The dermis demonstrated the inflammatory healing phase formed of few amounts of fibrous tissue (highlighted by the Masson’s trichrome stain ×100) 7.8±3.5%, an increased amount of granulation tissue formed of newly formed blood vessels 40±1.6/HPF, myofibroblasts 32±6.3/HPF (highlighted by the immunohistochemistry for α-SMA, ×400), and the number of lymphocytes 40±3.7/HPF (highlighted by the immunohistochemistry for CD-45, ×400). Skin specimen from multiple phage doses treatment (group 4) showed epidermal re-epithelialization of thick epidermis 61.2±1.2 μm with the regeneration of the skin appendages ≥5/wound area (score 3), (evidenced by H&E staining ×100). The dermis demonstrated the remodeling phase of wound healing formed mainly of collagen fibers 99±0.6% (highlighted by the Masson’s trichrome stain ×100) and a minimal amount of granulation tissue formed of newly formed blood vessels 2±0.4/HPF, myofibroblasts 1.4±0.3/HPF (highlighted by the immunohistochemistry for α-SMA, ×400), and lymphocytes 3±0.3/HPF (highlighted by the immunohistochemistry for CD-45, ×400).

Skin specimen from single phage dose treatment (group 5) displayed the epidermal re-epithelialization of thick epidermis 58±1.8 μm with the regeneration of the skin appendages ≥5/wound area (score 3) (evidenced by H&E staining ×100). The dermis was in the remodeling phase of wound healing, formed mainly of collagen fibers (highlighted by the Masson’s trichrome stain ×100) 97.3±2.3%, and a minimal amount of granulation tissue formed of newly formed blood vessels, 4±1/HPF, myofibroblasts 3±1.4/HPF, (highlighted by the immunohistochemistry for α-SMA, ×400), and lymphocytes 5±2.2/HPF (highlighted by the immunohistochemistry for CD-45, ×400).

## Discussion

Due to the misuse of antibiotics in clinical therapy, bacteria have evolved resistance against most commercial antibiotics. Many studies have demonstrated that phages are one of the most potent weapons for eradicating *P. aeruginosa* biofilms and planktonic cells due to their natural abundance, specificity, and safety [65].

This study isolated 50 clinical isolates of *P. aeruginosa* from Egyptian hospitalized patients and screened for antibiotic sensitivity. Their antibiotic profile indicated that the isolated *P. aeruginosa* were more resistant to levofloxacin than ciprofloxacin, which agreed with Zhao et al. [66]. FQs are the most commonly used antibiotics against *P. aeruginosa* infections; however*, P. aeruginosa* acquires resistance to the FQs through overexpression of efflux pumps and point mutations in target site genes [67]. Moreover, the tested *P. aeruginosa* isolates exhibited high resistance to β-lactams because of β-lactamase production, especially the AmpC β-lactamase, efflux pump expression, permeability changes, and changes in the target site [5, 68]. Besides, the *P. aeruginosa* isolates revealed high resistance to gentamicin, which agreed with a previous study that showed high aminoglycoside resistance among MDR and extensively drug-resistant (XDR) *P. aeruginosa* clinical isolates in Egypt [69]. Another study showed that the primary mechanism of resistance against aminoglycoside is methyltransferases responsible for changing the drug-binding sites [70]. Accordingly, 29 of the tested *P. aeruginosa* isolates in this study were MDR bacteria [71]. Seven virulence and antibiotic resistance of the tested *P. aeruginosa* isolates were confirmed using PCR, involving *Exo A*, *Exo S*, *Phz I and II*, *bla-TEM*, *OprL*, and *plcH*. Exo A gene is exotoxin A, which is responsible for local tissue damage and invasion of infection [72]. At the same time, the production of *Exo S* was recently linked to chronic infections as it causes cell necrosis, inhibition of DNA synthesis, and endocytosis [73]. In addition, *PhzI* and *PhzII* are required for pyocyanin production, which destroys the host cells and is essential for biofilm formation [74].

Moreover, the *Bla-TEM* gene indicates β-lactam resistance [75]. *OprL* is an essential component of the outer membrane of the bacterial cell, which maintains its integrity and is considered a marker for identifying *P. aeruginosa*-associated infections [76]. In addition, *PlcH* encodes a hemolytic toxin that destroys cell membranes and phospholipids [77]. According to the antibiotic profile and virulence genes, *P.s* 12 was chosen as a multi-drug resistant bacterial host for phage isolation and further studies. The phage ZCPA1 was isolated from raw sewage water sampled from Giza, Egypt, and both morphological analysis by TEM and genome sequencing confirmed that it belongs to the *Siphoviridae* family. The host range, physical stability against harsh environmental conditions, and lytic activity against planktonic and biofilm biomass of potential therapeutic phages are critical for ensuring their efficacy in being a good therapeutic agent. The potential phage for therapy must withstand a wide range of pH and temperature conditions in clinical and environmental applications [78]. The phage ZCPA1 exhibited a broad host range, which could infect 58% of the tested resistant *P. aeruginosa* clinical strains (*n* = 50). In addition, it showed good stability at a wide range of temperatures (37, 50, 60, and 70 °C) and a wide range of pH values (4–10). According to De Plano et al., the capsid structure of phages has been proposed for their resistance to harsh physical and chemical conditions, including pH and heat [79]. Besides, the reduction in phage titer at harsh pH conditions is due to the aggregation of viral particles, as shown by Langlet et al. [80]. Also, the phage ZCPA1 showed stability against UV light, which could be due to its linear double-stranded DNA. The type and organization of the phage genome can significantly affect the resistance of phage to UV inactivation. For example, phages with circular single-stranded DNA are more sensitive than those with linear single-stranded RNA. Those with linear double-stranded DNA exhibited the highest photoreactivation following UV exposure [81, 82].

Regarding the replication dynamics of phage ZCPA1, the one-step curve indicated that the latent period was about 90 min that could be relatively long compared to *Pseudomonas* phage DRL-P1 in a study by Sharma et al. [83]. Therefore, it could be explained that bacterial density is inversely proportional to the latent phage period since the low bacterial concentration led to a long phage latent period [84]. In this study, the ability of the phage to reduce the bacterial growth of *P.s* 12 was tested by a time-killing curve at different MOIs (0.1, 1, 10, 100). Based on the obtained results, the MOI 0.1 exhibited a negligible reduction in bacterial growth (*p* > 0.05), while starting from MOI 1, there was a significant bacterial reduction (*p* < 0.01). The highest level of bacterial reduction was observed at MOIs 10 and 100 (*p* < 0.001).

*P. aeruginosa* biofilm formation is a critical factor in pathogenicity and antimicrobial resistance [83]. It is also considered critical in delaying wound healing and leading to chronic wound infections [85]. As a result, the lytic activity of the phage ZCPA1 was evaluated against the established biofilm by *P.s* 12. Accordingly, at MOI 0.01, there was an insignificant reduction in the bacterial biofilm biomass (*p* > 0.05). However, at MOIs 0.1, 1, 10, and 100, there was about a 95% reduction in bacterial biofilm biomass (*p* < 0.001). According to these data, the phage ZCPA1 can significantly eradicate *P. aeruginosa* planktonic cells and biofilms. These findings agreed with previous studies [25]. According to Adnan et al., after 6-h treatment with MA-1 phage, it exhibited a significant (99.9%) reduction of 74-h-old biofilms compared to control [86]. In another study, the researchers isolated the AZ1 phage and tested its anti-biofilm activity, and the results showed about 99.9% reduction of 48-h-old biofilm biomass compared to the control [87]. As a result, the phages could have significant anti-biofilm activity. The ability of the phage to break down the biofilm structure is due to encoding a wide range of enzymes, including depolymerases and lysins. The depolymerases can specifically bind and degrade the exopolysaccharide of the host bacterial cells, disturbing the biofilm to facilitate the phage penetration to the bacterial cells. In addition, lysins are hydrolases encoded in the late stage of the lytic cycle of phage infection. They break down the peptidoglycans in the bacterial cell wall, releasing phage progenies from host cells and causing lysis and death [26].

In this study, the therapeutic efficiency of phage ZCPA1 was assessed on rats to treat full-thickness wounds infected with *P.s* 12. The wound closure percentage, bacterial count, and histopathological analysis throughout 17 days indicate that the phage ZCPA1 could be a promising antibacterial agent against the MDR *P. aeruginosa* with high-quality wound healing in a full-thickness wound infection in a rat model. Multiple research studies conducted in vivo experiments to study phages’ therapeutic effect and safety against a wide range of MDR bacteria. They exhibited significant results for killing the bacteria with minimal side effects [88–90]. Moreover, the efficacy of phage therapy against MDR *P. aeruginosa* in animal models was investigated. The researchers showed that phage could lead to a complete reduction of the bacterial count, inhibition of biofilm formation, and improvement of wound healing [25, 28, 34–37, 88, 91, 92].

Consequently, the influence of a topical application of phage ZCPA1, administered as single or multiple doses versus gentamicin antibiotic, was investigated in a rat model with *P. aeruginosa*-infected full-thickness excision wound. Skin regeneration, including restoring tissue integrity and function, is the most challenging aspect of wound treatment, as shown by Tottoli et al. [93]. However, the results in this study were promising as photo documentation and bacterial count demonstrated that the phage administration, either single-dose or multi-dose, resulted in 100% bacterial eradication and wound closure. Furthermore, the equivalent results between single-dose and multi-dose phage treatment underlined its self-replicating nature in the presence of susceptible microorganisms, a highly desired characteristic for any antibacterial drug [94].

Furthermore, re-epithelization is critical to successful wound healing [95]. According to the results of our histopathological analysis, both the single-dose and multiple-dose phage-treated rats showed epidermal re-epithelialization of the thick epidermis with the regeneration of the skin appendage similar to the uninfected-wound rats (positive control). In contrast, the gentamicin-treated rats showed epidermis ulceration, lacked skin appendages, and the infected rats without treatment (negative control) exhibited extensive epidermal ulceration and crust formation without skin appendages. According to Yousefpour et al., exposure of the *P. aeruginosa* to the sub-MIC dose of gentamicin showed increased bacterial virulence due to the antibiotic stress [96]. As the infected wound showed a healing delay in the infected group without treatment and the gentamicin-treated group, bacterial resistance can develop significant wound delay with serious complications. Moreover, we recommend extending this experiment to evaluate the efficacy of antibiotics to which the bacterial strain is sensitive, for example, imipenem, as the positive control. In addition, we can study the synergetic effect of phage when combined with two types of antibiotics, one to which the bacterial strain is sensitive and another is resistant as recent publications are recommending the combination therapy [97, 98].

The results of this study have shown the potential of phage for controlling full-thickness excision wounds caused by *P. aeruginosa.* It is recommended for further studies to study the antibiofilm ability of the phage against established biofilm by *P. aeruginosa* on the wound-infected rat model.

## Conclusions

The emergence of antibiotic resistance has led researchers to look for a safe and effective alternative to antibiotics. Phage therapy has exhibited the potential to control the MDR bacteria, such as *P. aeruginosa*. In this study, a novel phage ZCPA1 was isolated and characterized in vitro against MDR *P. aeruginosa*. ZCPA1 is a member of the *Siphoviridae* family, with a wide host range and high temperature, pH, and UV stability. Moreover, it has high lytic activity against MDR *P. aeruginosa* in planktonic and biofilms. The therapeutic efficacy of ZCPA1 was investigated in a full-thickness wound infected with *P. aeruginosa* in a rat model. *P. aeruginosa* inoculated wounds treated with phage showed 100% wound closure, high-quality regeneration, and complete bacterial infection elimination. Therefore, all these properties make phage ZCPA1 a promising therapeutic agent against *P. aeruginosa* skin wound infections*.* However, more research is required on the phage formulations to be used topically on wound infections and tested in clinical trials.

## Supplementary Information

**Additional file 1: Table S1.** Antimicrobial susceptibility test for *P. aeruginosa* isolates over eight different antibiotics. **Table S2.** Genome annotation of the ZCPA1 genome. **Figure S1.** The genetic map of the phage ZCPA1.

## Data Availability

The dataset presented in this study can be found in NCBI GenBank. The 16s rRNA sequence was deposited under the accession number OL375153. In addition, the annotated genome sequence of the phage ZCPA1was deposited under the accession numbers OL597541:OL597590.

## Abbreviations

MDR: Multi-drug resistant
MOI: Multiplicity of infection
CFU/mL: Colony forming unit/mL
PFU/mL: Plaque forming unit/mL
IC: Phage infective center
IHC: Immunohistochemical
