# Supplementary material for: Bacteriophage as a potential therapy to control antibiotic-resistant Pseudomonas aeruginosa infection through topical application onto a full-thickness wound in a rat model
Source: J Genet Eng Biotechnol. 2022 Sep 12;20:133. doi: 10.1186/s43141-022-00409-1 (PMC9468208; doi:10.1186/s43141-022-00409-1)
Supplement: Supplementary file 1 — Additional file 1: Table S1. Antimicrobial susceptibility test for P. aeruginosa isolates over eight different antibiotics. Table S2. Genome annotation of the ZCPA1 genome. Figure S1. The genetic map of the phage ZCPA1. [file 43141_2022_409_MOESM1_ESM.docx]

| Antibiotics | Fluoroquinolones | | β-lactam | | | Aminoglycoside | | Cephalosporin | MAR index |
| --- | --- | --- | --- | --- | --- | --- | --- | --- | --- |
|  | **Levofloxacin** | **Ciprofloxacin** | **Piperacillin** | **Imipenem** | **Meropenem** | **Gentamicin** | **Amikacin** | **Cefepime** |  |
| *P. s* 1 | S | R | R | S | S | R | S | S | 0.37 |
| *P. s* 2 | R | R | R | R | R | R | I | R | 0.88 |
| *P. s* 3 | S | S | S | S | S | S | S | S | 0 |
| *P. s* 4 | R | R | I | S | R | R | S | R | 0.63 |
| *P. s* 5 | R | R | I | S | R | R | S | R | 0.63 |
| *P. s* 6 | R | R | I | S | R | R | S | R | 0.63 |
| *P. s* 8 | R | R | S | R | R | R | S | S | 0.63 |
| *P. s* 9 | R | R | I | R | R | R | R | R | 0.88 |
| *P. s* 10 | R | R | R | I | R | R | R | R | 0.88 |
| *P. s* 11 | R | R | I | R | R | R | R | R | 0.88 |
| *P. s* 12 | R | R | I | S | R | R | R | I | 0.63 |
| *P. s* 13 | S | I | S | S | S | I | S | S | 0 |
| *P. s* 14 | R | R | I | R | R | R | R | R | 0.88 |
| *P. s* 15 | S | S | S | S | R | S | S | S | 0.13 |
| *P. s* 16 | S | S | S | S | R | S | S | S | 0.13 |
| *P. s* 17 | R | S | S | S | R | S | S | S | 0.25 |
| *P. s* 18 | I | S | S | S | S | S | S | S | 0.13 |
| *P. s* 19 | S | S | S | S | S | S | S | S | 0 |
| *P. s* 20 | S | S | S | S | S | S | S | S | 0 |
| *P. s* 21 | R | R | S | R | R | S | S | I | 0.63 |
| *P. s* 22 | S | I | S | S | S | S | S | S | 0.13 |
| *P. s* 23 | S | S | S | S | S | S | S | S | 0 |
| *P. s* 24 | I | S | S | S | S | S | S | S | 0.13 |
| *P. s* 25 | S | S | S | S | S | S | S | S | 0 |
| *P. s* 26 | I | I | S | S | S | S | S | S | 0.25 |
| *P. s* 27 | S | S | S | S | R | R | S | R | 0.38 |
| *P. s* 28 | S | I | S | S | S | S | S | S | 0.13 |
| *P. s* 29 | I | S | S | S | S | S | S | S | 0.13 |
| *P. s* 30 | I | I | I | S | S | S | S | R | 0.5 |
| *P. s* 31 | I | I | S | S | S | S | S | S | 0.25 |
| *P. s* 32 | R | R | R | R | R | R | R | R | 1 |
| *P. s* 33 | R | R | S | R | R | R | R | R | 0.9 |
| *P. s* 34 | R | R | R | R | R | R | R | R | 1 |
| *P. s* 35 | R | R | R | R | R | R | R | R | 1 |
| *P. s* 36 | R | R | R | R | R | R | R | R | 1 |
| *P. s* 37 | R | R | R | R | R | R | R | R | 1 |
| *P. s* 38 | R | S | S | R | R | S | S | S | 0.38 |
| *P. s* 39 | R | R | R | R | R | R | R | R | 1 |
| *P. s* 40 | R | R | R | R | R | R | R | R | 1 |
| *P. s* 41 | R | R | R | R | R | R | R | R | 1 |
| *P. s* 42 | R | R | R | R | R | R | R | R | 1 |
| *P. s* 43 | R | R | R | R | R | R | R | R | 1 |
| *P. s* 44 | R | R | R | R | R | R | R | R | 1 |
| *P. s* 45 | R | S | S | R | I | S | R | S | 0.5 |
| *P. s* 46 | R | R | R | R | R | R | S | R | 0.9 |
| *P. s* 47 | R | R | R | R | R | R | R | R | 1 |
| *P.s* 48 | R | R | R | R | R | R | R | R | 1 |
| *P.s* 49 | R | R | R | R | R | R | R | R | 1 |
| *P.s* 50 | R | R | R | R | R | R | R | R | 1 |
| R% | 65 % | 59% | 38% | 51% | 67% | 63% | 44% | 55% |  |

Table S1: Antimicrobial susceptibility test for P. aeruginosa isolates over eight different antibiotics.

The abbreviations are as follows: (S) Sensitive, (I): Intermediate, (R) Resistant, and MAR: Multi-drug resistant.

*Table S2: Genome annotation of the ZCPA1 genome*

| **ORF number** | **Strand** | **Frame** | **CDS position** | **Translation** | **Best annotated protein** |
| --- | --- | --- | --- | --- | --- |
| lcl\|ORF1:10:156 | + | 1 | 10 .. 156 | 147 \| 48 | Hypothetical protein |
| lcl\|ORF1:13:177 | + | 1 | 13 .. 177 | 165 \| 54 | Membrane protein |
| lcl\|ORF1:187:648 | + | 1 | 187 .. 648 | 462 \| 153 | Hypothetical protein |
| lcl\|ORF1:19:270 | + | 1 | 19 .. 270 | 252 \| 83 | Hypo thetical protein |
| lcl\|ORF1:22:102 | + | 1 | 22 .. 102 | 81 \| 26 | Hypothetical protein |
| lcl\|ORF1:28:432 | + | 1 | 28 .. 432 | 405 \| 134 | Terminase large subunit |
| lcl\|ORF1:31:744 | + | 1 | 31 .. 744 | 714 \| 237 | Minor capsid protein |
| lcl\|ORF1:409:690 | + | 1 | 409 .. 690 | 282 \| 93 | Hypothetical protein |
| lcl\|ORF1:46:486 | + | 1 | 46 .. 486 | 441 \| 146 | Hypothetical protein |
| lcl\|ORF1:7:504 | + | 1 | 7 .. 504 | 498 \| 165 | Hypothetical protein |
| lcl\|ORF10:12:278 | + | 3 | 12 .. 278 | 267 \| 88 | Hypothetical protein |
| lcl\|ORF10:888:499 | - | 2 | 888 .. 499 | 390 \| 129 | Hypothetical protein |
| lcl\|ORF11:1151:750 | - | 1 | 1151 .. 750 | 402 \| 133 | Hypothetical protein |
| lcl\|ORF11:229:2 | - | 2 | 229 .. >2 | 228 \| 75 | Hypothetical protein |
| lcl\|ORF11:282:779 | + | 3 | 282 .. 779 | 498 \| 165 | Hypothetical protein |
| lcl\|ORF11:545:739 | + | 2 | 545 .. 739 | 195 \| 64 | Hypothetical protein |
| lcl\|ORF11:753:466 | - | 3 | 753 .. 466 | 288 \| 95 | Hypothetical protein |
| lcl\|ORF12:281:628 | + | 2 | 281 .. 628 | 348 \| 115 | Hypothetical protein |
| lcl\|ORF12:366:1 | - | 3 | 366 .. >1 | 366 \| 121 | Hypothetical protein |
| lcl\|ORF12:781:476 | - | 2 | 781 .. 476 | 306 \| 101 | Tape measure protein |
| lcl\|ORF12:906:2174 | + | 3 | 906 .. >2174 | 1269 \| 422 | Tape measure protein |
| lcl\|ORF13:1305:1 | - | 2 | 1305 .. >1 | 1305 \| 434 | Tape measure protein |
| lcl\|ORF13:551:3 | - | 1 | 551 .. >3 | 549 \| 182 | Hypothetical protein |
| lcl\|ORF14:979:344 | - | 1 | 979 .. 344 | 636 \| 211 | DNA-binding domain-containing protein |
| lcl\|ORF14:992:1459 | + | 2 | 992 .. 1459 | 468 \| 155 | Structural protein |
| lcl\|ORF15:1335:2609 | + | 3 | 1335 .. >2609 | 1275 \| 424 | Virion structural protein |
| lcl\|ORF15:897:529 | - | 1 | 897 .. 529 | 369 \| 122 | Hypothetical protein |
| lcl\|ORF16:1679:1239 | - | 3 | 1679 .. 1239 | 441 \| 146 | Protease |
| lcl\|ORF16:1983:2570 | + | 3 | 1983 .. >2570 | 588 \| 195 | Minor capsid protein |
| lcl\|ORF16:468:1 | - | 3 | 468 .. >1 | 468 \| 155 | Virion structural protein |
| lcl\|ORF16:480:226 | - | 1 | 480 .. 226 | 255 \| 84 | Hypothetical protein |
| lcl\|ORF17:1235:321 | - | 3 | 1235 .. 321 | 915 \| 304 | Mu-like promajor head subunit gpt family protein |
| lcl\|ORF17:2096:2398 | + | 2 | 2096 .. 2398 | 303 \| 100 | Hypothetical protein |
| lcl\|ORF17:999:577 | - | 3 | 999 .. 577 | 423 \| 140 | Hypothetical protein |
| lcl\|ORF18:2888:3454 | + | 2 | 2888 .. 3454 | 567 \| 188 | Hypothetical protein |
| lcl\|ORF18:302:3 | - | 3 | 302 .. >3 | 300 \| 99 | Hypothetical protein |
| lcl\|ORF19:1940:1587 | - | 1 | 1940 .. 1587 | 354 \| 117 | Virion morphogenesis protein |
| lcl\|ORF2:35:235 | + | 2 | 35 .. 235 | 201 \| 66 | Hypothetical protein |
| lcl\|ORF2:35:346 | + | 2 | 35 .. 346 | 312 \| 103 | Family transposase |
| lcl\|ORF2:467:622 | + | 2 | 467 .. 622 | 156 \| 51 | Hypothetical protein |
| lcl\|ORF2:538:1017 | + | 1 | 538 .. >1017 | 480 \| 159 | PF10983 family protein |
| lcl\|ORF20:1475:1053 | - | 1 | 1475 .. 1053 | 423 \| 140 | Anti-CRISPR protein acrf3 |
| lcl\|ORF21:573:1043 | + | 3 | 573 .. 1043 | 471 \| 156 | Hypothetical protein |
| lcl\|ORF23:1527:2099 | + | 3 | 1527 .. 2099 | 573 \| 190 | Hypothetical protein |
| lcl\|ORF23:2855:2406 | - | 1 | 2855 .. 2406 | 450 \| 149 | Virion structural protein |
| lcl\|ORF23:347:3 | - | 3 | 347 .. >3 | 345 \| 114 | Hypothetical protein |
| lcl\|ORF25:3261:3389 | + | 3 | 3261 .. 3389 | 129 \| 42 | Hypothetical protein |
| lcl\|ORF26:3411:3836 | + | 3 | 3411 .. 3836 | 426 \| 141 | Hypothetical protein |
| lcl\|ORF27:1586:1347 | - | 1 | 1586 .. 1347 | 240 \| 79 | Hypothetical protein |
| lcl\|ORF29:1196:3 | - | 1 | 1196 .. >3 | 1194 \| 397 | Tail protein |
| lcl\|ORF29:3553:2909 | - | 1 | 3553 .. 2909 | 645 \| 214 | Hypothetical protein |
| lcl\|ORF3:1024:1428 | + | 1 | 1024 .. 1428 | 405 \| 134 | Virion structural protein |
| lcl\|ORF3:14:619 | + | 2 | 14 .. 619 | 606 \| 201 | Tail protein |
| lcl\|ORF3:5:499 | + | 2 | 5 .. >499 | 495 \| 164 | Terminase large subunit |
| lcl\|ORF3:958:1257 | + | 1 | 958 .. >1257 | 300 \| 99 | Hypothetical protein |
| lcl\|ORF31:2416:1805 | - | 2 | 2416 .. 1805 | 612 \| 203 | BR0599 family protein |
| lcl\|ORF32:1059:835 | - | 3 | 1059 .. 835 | 225 \| 74 | Winged helix-turn-helix domain-containing protein |
| lcl\|ORF32:1768:1595 | - | 2 | 1768 .. 1595 | 174 \| 57 | BR0599 family protein |
| lcl\|ORF33:615:1 | - | 3 | 615 .. >1 | 615 \| 204 | Protease |
| lcl\|ORF39:3204:3121 | - | 2 | 3204 .. 3121 | 84 \| 27 | Hypothetical protein |
| lcl\|ORF4:173:691 | + | 2 | 173 .. 691 | 519 \| 172 | Host-nuclease inhibitor Gam family protein |
| lcl\|ORF4:185:616 | + | 2 | 185 .. 616 | 432 \| 143 | Hypothetical protein |
| lcl\|ORF4:204:428 | + | 3 | 204 .. >428 | 225 \| 74 | Helix-turn-helix domain-containing protein |
| lcl\|ORF4:372:500 | + | 3 | 372 .. 500 | 129 \| 42 | Hypothetical protein |
| lcl\|ORF4:397:2 | - | 1 | 397 .. >2 | 396 \| 131 | Terminase large subunit |
| lcl\|ORF4:435:1 | - | 1 | <435 .. >1 | 435 \| 144 | Response regulator |
| lcl\|ORF4:78:410 | + | 3 | 78 .. 410 | 333 \| 110 | Helix-turn-helix transcriptional regulation |
| lcl\|ORF40:1467:1132 | - | 3 | 1467 .. 1132 | 336 \| 111 | Hypothetical protein |
| lcl\|ORF5:539:3 | - | 1 | 539 .. >3 | 537 \| 178 | Tape measure protein |
| lcl\|ORF5:744:848 | + | 3 | 744 .. >848 | 105 \| 34 | Virion morphogenesis protein |
| lcl\|ORF5:87:203 | + | 3 | 87 .. 203 | 117 \| 38 | Hypothetical protein |
| lcl\|ORF6:1261:1701 | + | 1 | 1261 .. 1701 | 441 \| 146 | Hypothetical protein |
| lcl\|ORF6:20:1483 | + | 2 | 20 .. 1483 | 1464 \| 487 | Mu transposase C-terminal domain-containing protein |
| lcl\|ORF6:351:1 | - | 2 | 351 .. >1 | 351 \| 116 | Replication protein P |
| lcl\|ORF6:381:34 | - | 3 | 381 .. 34 | 348 \| 115 | PGM-like protein |
| lcl\|ORF6:579:746 | + | 3 | 579 .. >746 | 168 \| 55 | Hypothetical protein |
| lcl\|ORF6:630:743 | + | 3 | 630 .. >743 | 114 \| 37 | Hypothetical protein |
| lcl\|ORF7:1951:2205 | + | 1 | 1951 .. 2205 | 255 \| 84 | Hypothetical protein |
| lcl\|ORF7:240:145 | - | 1 | 240 .. 145 | 96 \| 31 | Hypothetical protein |
| lcl\|ORF7:374:258 | - | 1 | 374 .. 258 | 117 \| 38 | Hypothetical protein |
| lcl\|ORF7:379:2 | - | 3 | <379 .. >2 | 378 \| 125 | ABC-type multidrug transport system, atpase and permease component |
| lcl\|ORF7:550:2 | - | 2 | 550 .. >2 | 549 \| 182 | Mu transposase C-terminal domain-containing protein |
| lcl\|ORF7:643:2 | - | 1 | 643 .. >2 | 642 \| 213 | Hypothetical protein |
| lcl\|ORF7:809:898 | + | 2 | 809 .. >898 | 90 \| 29 | Hypothetical protein |
| lcl\|ORF8:617:904 | + | 2 | 617 .. 904 | 288 \| 95 | Hypothetical protein |
| lcl\|ORF8:693:974 | + | 3 | 693 .. 974 | 282 \| 93 | Host nuclease inhibitor protein |
| lcl\|ORF9:111:1682 | + | 3 | 111 .. >1682 | 1572 \| 523 | Hypothetical protein |
| lcl\|ORF9:419:1165 | + | 2 | 419 .. 1165 | 747 \| 248 | Virion structural protein |
| lcl\|ORF9:483:812 | + | 3 | 483 .. 812 | 330 \| 109 | Hypothetical protein |
| lcl\|ORF9:937:113 | - | 1 | 937 .. 113 | 825 \| 274 | Hypothetical protein |


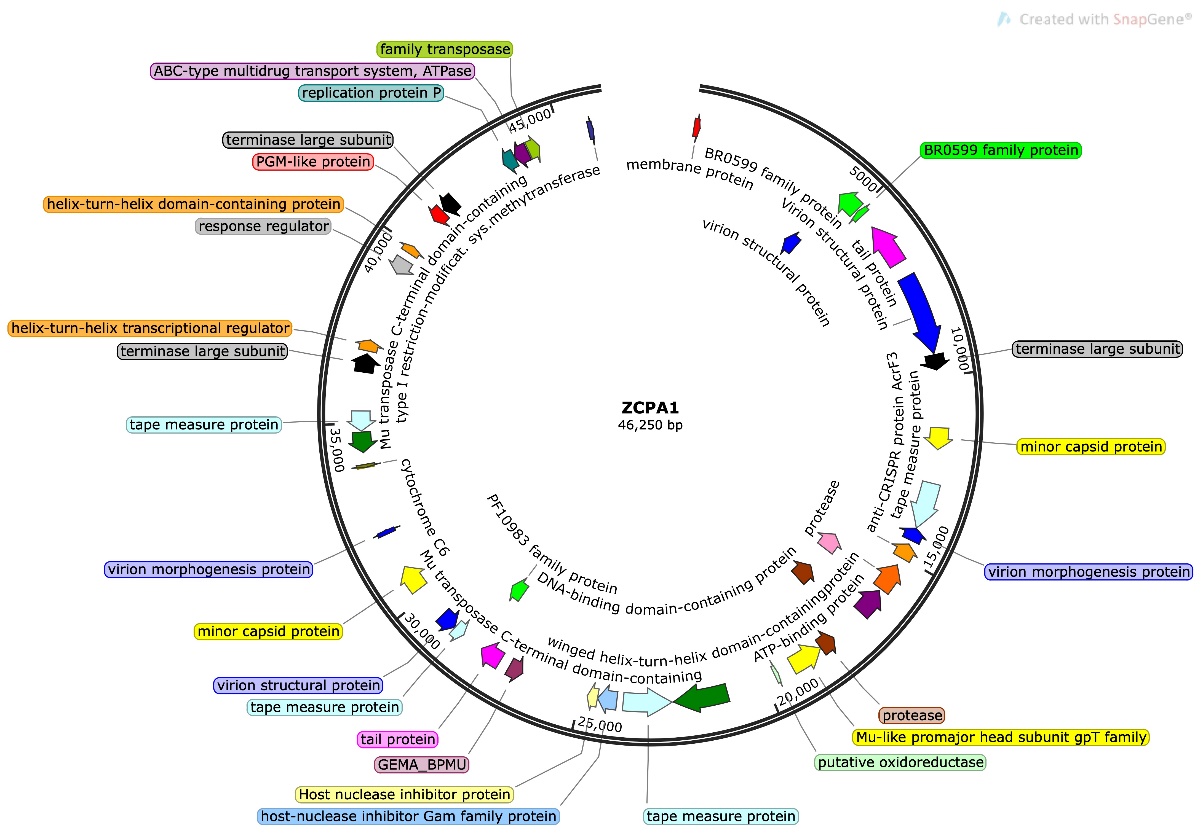


Figure S1. The genetic map of the phage ZCPA1
